# Strategy mediation in working memory training in younger and older adults

**DOI:** 10.1177/1747021820915107

**Published:** 2020-04-23

**Authors:** Alicia Forsberg, Daniel Fellman, Matti Laine, Wendy Johnson, Robert H Logie

**Affiliations:** 1 Department of Psychology, The University of Edinburgh, Edinburgh, UK; 2 The University of Missouri, Columbia, MO, USA; 3 Department of Psychology, Åbo Akademi University, Turku, Finland; 4 Department of Applied Educational Science, Umeå University, Umeå, Sweden

**Keywords:** Working memory, cognitive training, N-back, cognitive ageing

## Abstract

Working memory (WM) training with the N-Back task has been argued to improve cognitive capacity and general cognitive abilities (the Capacity Hypothesis of training), although several studies have shown little or no evidence for such improvements beyond tasks that are very similar to the trained task. Laine et al. demonstrated that instructing young adult participants to use a specific visualisation strategy for N-back training resulted in clear, generalised benefits from only 30 min of training (Strategy Mediation Hypothesis of training). Here, we report a systematic replication and extension of the Laine et al. study, by administering 60 younger and 60 older participants a set of WM tasks before and after a 30-min N-back training session. Half the participants were instructed to use a visualisation strategy, the others received no instruction. The pre-post test battery encompassed a criterion task (digit N-back), two untrained tasks N-back tasks (letters and colours), and three structurally different WM tasks. The instructed visualisation strategy significantly boosted at least some measures of N-back performance in participants of both age groups, although the strategy generally appeared more difficult to implement and less beneficial for older adults. However, the strategy did not improve performance on structurally different WM tasks. We also found significant associations between N-back performance and the type and level of detail of self-generated strategies in the uninstructed participants, as well as age group differences in reported strategy types. WM performance appeared to partly reflect the application of strategies, and Strategy Mediation should be considered to understand the mechanisms of WM training. Claims of efficient training should demonstrate useful improvement beyond task-specific strategies.

Working memory (WM) refers to cognitive functions that support the ready availability of a small amount of information on a temporary basis while we undertake ongoing actions and mental activities (e.g., Logie & Cowan, 2015). WM is viewed as a core mechanism underpinning higher-order cognitive abilities such as perception and problem-solving (Ma et al., 2014), and is related to fluid intelligence (Kane et al., 2005; Unsworth et al., 2014), reasoning ability (Conway et al., 2003; Kyllonen & Christal, 1990), and measures of cognitive control (Conway et al., 2001; Kane & Engle, 2003; Redick et al., 2011). WM also suffers pronounced, linear decline during adult ageing (Bopp & Verhaeghen, 2005; Borella et al., 2008; Park & Payer, 2006), although some aspects of WM decline faster than others; verbal WM appears least susceptible, and visuo-spatial most susceptible to age-related decline (Johnson et al., 2010; Park et al., 2002). Functioning of WM abilities is important for the autonomy and well-being in older adults (Tomaszewski Farias et al., 2009). Hence, when early studies suggested that repeated adaptive WM training could protect older adults from cognitive decline (e.g., Brehmer et al., 2012), there was great interest (Green & Bavelier, 2008; Klingberg, 2010; Lövdén et al., 2010; Morrison & Chein, 2011; von Bastian & Oberauer, 2014), due to the potential benefits to public health and well-being. In addition, several commercial companies have promoted WM training software, claiming scientific support for a range of benefits such as an increasing IQ (Mindsparke, 2011), improving grades (Jungle Memory, 2011), and reducing day-to-day lapses of attention (Cogmed, 2011).

Contemporary approaches to cognitive training stem from evidence of neural plasticity related to cognition in both younger and older adults (e.g., Hertzog et al., 2008). The brain was likened to muscles, growing physically larger and stronger when repeatedly challenged at close to maximum currently manageable difficulty (i.e., adaptivity). Based on this analogy, researchers proposed that such challenging training of WM increases WM capacity (e.g., Morrison & Chein, 2011) by eliciting functional and anatomical changes in the brain (Dahlin et al., 2008). Such changes, they suggested, may help preserve brain integrity as we age, and produce lasting improvements in fluid intelligence, if WM and fluid intelligence rely on a shared capacity constraint (Halford et al., 2007). The attractive idea of increased WM capacity as a result of training has been referred to as the *Capacity Hypothesis* of WM training (Peng & Fuchs, 2017). If training improves cognitive functioning (capacity) beyond the performance of the trained task, training benefits should generalise to other cognitive tasks due to the strong relationship between WM and other cognitive activities (e.g., Daneman & Merikle, 1996). The distinction between near- and far-transfer (see the taxonomy proposed by Noack et al., 2009; see also Karbach & Kray, 2009) is therefore crucial to the debate on the efficacy of WM training. Near-transfer indicates improvements on tasks very similar to the trained task itself. In contrast, to demonstrate far-transfer, WM training should improve performance on, for example, measures of fluid intelligence, or reasoning tasks that, crucially, are quite unlike the trained task. Recently, some authors (e.g., de Simoni & von Bastian, 2018; Soveri et al., 2017) have separated the near-transfer domain into two categories according to the similarity of the tasks to the trained WM task, namely task-specific near-transfer and task-general near-transfer. Task-specific near-transfer refers to improvements in WM tasks sharing the same task paradigm with the trained task, whereas task-general near-transfer refers to improvements in WM tasks that are structurally dissimilar to the trained task. Failure to separate these two types of near-transfer might make near-transfer effects seem broader than they actually are (see Soveri et al., 2017 for a re-analysis of Melby-Lervåg et al., 2016), or may obscure task-specific near-transfer effects. In this article, we investigated how task strategies that are developed or used even in a single brief training session may influence task-specific near-transfer effects.

While WM training initially appeared promising (training improved performance even on untrained, quite different cognitive tasks in healthy adults: Jaeggi et al., 2008, and children with attention-deficit hyperactivity disorder [ADHD]; Klingberg et al., 2002), subsequent research in healthy children and younger adults challenged these claims. With more appropriate experimental controls, it appeared that WM training typically improved performance on the trained task itself, as well as on other verbal and visuospatial WM tasks that were similar to the trained task, whereas far-transfer effects to reasoning, or fluid intelligence were at most small and unreliable across different studies (for comprehensive meta-analyses, see Melby-Lervåg et al., 2016; Melby-Lervåg & Hulme, 2013; Schwaighofer et al., 2015; Weicker et al., 2016; see also Dougherty et al., 2016; Kassai et al., 2019; Lampit et al., 2014; McCabe et al., 2016; Redick, 2019; Simons et al., 2016). Evidence regarding the effects of training in older adults is also mixed. A meta-analysis of 13 studies indicated that WM training in healthy older adults produced both large near- and far-transfer effects (Karbach & Verhaeghen, 2014). However, when Melby-Lervåg et al. (2016) replicated the meta-analysis by only including studies which compared the trained group to active controls and controlled for baseline differences, they found much smaller effects of training than originally reported. Moreover, in a recent meta-analysis of the commonly used N-back WM training by Soveri et al. (2017), the only more substantial effects following WM training were seen in task-specific near-transfer measures, that is, in tasks that were structurally similar to the trained WM task(s). In general, meta-analyses with less stringent inclusion criteria typically find both near- and far-transfer effects in older adults (e.g., Chiu et al., 2017). It has been difficult to reach consensus regarding the effects of cognitive training due to variations in training paradigms and in what is considered an appropriate control group (see Melby-Lervåg et al., 2016; Melby-Lervåg & Hulme, 2013; Morrison & Chein, 2011; Shipstead et al., 2010, 2012).

In addition to methodological inconsistencies, different theoretical perspectives may contribute to confusion in the literature. Some theories propose that online cognition is limited by the capacity of a domain-general attentional resource or WM system (Engle & Kane, 2004), and advocates for the benefits of WM training argue that this resource can be increased by WM training, thus enhancing general cognitive abilities (Au et al., 2015; Jaeggi et al., 2008). For example, the amount of information WM can retain and manipulate is thought to constrain “fluid” intelligence, as measured by Raven’s Progressive Matrices (Jaeggi et al., 2008). According to the *Capacity Hypothesis* of WM training (Peng & Fuchs, 2017) cited above, WM training should improve a general mental WM workspace, and thus perhaps result in improved performance on such measures of “fluid” intelligence.

In contrast, other theories view WM as involving a variety of cognitive systems, among which participants select according to task demands (Baddeley & Logie, 1999; Logie, 2011; Logie & Niven, 2012). For instance, one system may retain phonological codes, another visual codes. When tasked to remember sets of digits, participants may remember them phonologically, by their visual shapes, or using a semantic memory strategy. Therefore, performance may reflect use of different cognitive resources in different participants (Johnson et al., 2010; Logie, 2018; Logie et al., 1996, 2011; Thurstone, 1931), and crucially, participants may change how they attempt to perform a task as they see how well a given strategy works with repeated trials, or as a result of explicit instruction. Training thus might improve one particular cognitive skill, or lead to strategic recruitment of a different cognitive mechanism, with potentially different implications for transfer to other tasks. Based on studies that had indicated improved Raven’s Matrices performance following training with the commercial Cogmed WM training programme (Roughan & Hadwin, 2011), Shipstead et al. (2012) suggested that this might occur because this test used to measure “fluid” intelligence requires visual processing and matching very similar to the tasks trained with Cogmed. Thus, WM training may improve specific abilities, rather than improving some underlying intelligence “capacity.” It is also possible that training results in development of highly practiced cognitive skills so that, after training, the tasks that require these skills rely less (or not at all) on WM capacity (e.g., Gopher et al., 1989; Schneider, 1985). In this argument, the capacity of WM previously required for the untrained task is then available for other tasks, giving the misleading impression that its capacity has increased (for discussions see Gathercole et al., 2019; Logie, 2012, 2018).

Typically, adaptive training (i.e., tasks get harder as the participant improves) is associated with significantly better performance improvement than non-adaptive training (i.e., performing the task at a consistent level of difficulty; for example, Holmes et al., 2009; Klingberg et al., 2005; Olson & Jiang, 2004; Thorell et al., 2009) and is seen as a key ingredient of effective training. Interestingly, some evidence suggests that adaptive training may also affect strategy use. Post-training interviews following Cogmed training indicated that participants in an adaptive training group reported using grouping strategies significantly more than did active and passive control group participants. This was associated with larger performance gains in some of the post-tests (Dunning & Holmes, 2014). This suggested that adaptive training may be comparatively more beneficial because participants are encouraged to develop new strategies as the task gets more challenging.

Laine et al. (2018) proposed and explicitly tested one aspect of this, the *Strategy Mediation Hypothesis* of WM training: that task-specific near-transfer gains are driven by developing and using a task-specific strategy during training. In younger adults, they used the N-back training paradigm (Kirchner, 1958) in which participants see an ongoing string of individual stimuli (e.g., digits) stream on a computer screen. They are asked to indicate whether each stimulus is identical to that presented *n* items back. Laine et al. (2018) instructed some young adult participants to use a particular visualisation strategy during a single 30-min N-back training session. This strategy instruction resulted in significant improvement in the trained N-back task (with digits), and in two untrained N-back tasks using different stimuli (i.e., letters or colours), compared to participants who received no strategy instruction. Furthermore, the level of detail and type of self-generated N-back strategies reported by the uninstructed participants was significantly related to their post-test N-back performance. The results in Laine et al. (2018) provided strong evidence for the Strategy Mediation Hypothesis, according to which strategy changes rather than increased WM capacity may underlie successful WM training outcomes (Dunning & Holmes, 2014; Soveri et al., 2017).

However, the Strategy Mediation and Capacity Hypotheses are not mutually exclusive. While associations of performance gains with strategies provide support for the Strategy Mediation Hypothesis, they do not rule out the possibility that training increases actual capacity of some sort. Laine et al.’s (2018) finding that practising with a strategy for 30 min resulted in gains equivalent to those typically observed after 5 weeks of N-back training did indicate that for training studies to be taken seriously, they should also demonstrate that trained participants developing a task-specific strategy cannot alone explain improved performance. For instance, the strategy of visualising digits used by Laine et al. may be unlikely to improve general reasoning or prevent age-related cognitive decline, but it did appear to boost N-back performance greatly. Establishing the mechanisms behind training-induced performance improvements is crucial to determining whether the intended cognitive improvement has occurred, and what factors might have led to any such improvement.

Moreover, important findings should be replicated, ideally in a different lab and with a different participant sample (see Simons, 2014). Therefore, in this study, we conducted a systematic replication of Laine et al. (2018) in a different country, using an online methodology, and unlike that previous study, also recruited healthy older adults.

Similar to the original study, our purpose was not to falsify the Capacity Hypothesis. Instead, we tested the Strategy Mediation Hypothesis by investigating the roles strategy use can play in these tasks, to further explore its role as one possible source for WM training outcomes. Specifically, our research question was: what are the effects of instructed and self-generated strategy use on WM updating performance, in healthy younger and older adults? We assessed this in the two age groups by testing the hypothesis that explicit instruction to use a visuospatial grouping and comparison strategy in a digit N-back task would improve performance in the trained task and in untrained N-back tasks employing different stimuli (letters, colours) in younger adults (directional; replication of findings in Laine et al., 2018; H_1_). Moreover, evidence suggests that older adults are not merely like poorly performing younger adults (e.g., Perfect & Maylor, 2000; Rabbitt, 2005). Instead, as noted earlier, different cognitive abilities appear to decline at different rates, and younger and older adults may use different cognitive resources when performing the same cognitive task (e.g., Johnson et al., 2010). Therefore, it is unclear whether Laine et al.’s (2018) visualisation strategy would be equally efficient in older adults, and whether non-instructed older adults would make different strategic choices than younger adults. However, healthy older adults are a target group for training, given that they might be worried about cognitive decline (e.g., Federal Trade Commission, 2016). So, it is important to discover whether or not such training packages are likely to be beneficial. Some previous studies instructing participants to apply mnemonic techniques or strategies have found more substantial training gains in younger than in older adults (e.g., Lövdén et al., 2012; Verhaeghen et al., 1992; Verhaeghen & Marcoen, 1996 but see Gross et al., 2012). However, older adults’ WM performance benefitted from instruction to switch to visual codes in a verbal WM task (Osaka et al., 2012). Due to a lack of background evidence on how this specific strategy actually improves performance, we hypothesised that explicit strategy instruction would affect post-test performance in healthy older adults to the same extent as in younger adults (H_2_). Next, we hypothesised that reported self-generated strategies (in the non-instructed group) would be associated with better memory performance on the trained N-back task and in untrained N-back tasks employing different stimuli (letters, colours) in younger adults (directional; replication of findings in Laine et al., 2018, H_3_), and that similar effects of self-generated strategies would be observed in the older adults as well (H_4_).

The four hypotheses, methods, and analyses were pre-registered via the Open Science Framework (https://osf.io/npzkc).

## Method

### Participants

Our pre-registered target sample size was 60 younger and 60 older adults. These numbers ensured a power of at least .95 to detect a medium effect of strategy condition on the trained N-back digit task, and a power of .80 to detect near-transfer to other N-back tasks, determined by a power analysis using G*power (Faul et al., 2007), based on effect sizes in the study we aimed to replicate (Laine et al., 2018).^1^ We recruited a total of 136 participants: 74 younger adults who were students or former students at the University of Edinburgh, and 62 older adults who were members of a Participant Volunteer Panel, or a lifelong learning group. Two older and 13 younger adults were excluded and replaced for failing to complete all three sessions. We excluded one younger participant who reported using pen and paper in the memory tasks, and one who completed the first session twice. The final sample consisted of 60 younger adults (*M* = 22.50, *SD* = 3.50 years), and 60 older adults (*M* = 69.30, *SD* = 5.46 years). All older adults had either scored above the recommended threshold for cognitive impairments (Addenbrooke’s Cognitive Examination [ACE-III]; Hodges & Larner, 2017; Mioshi et al., 2006) within 2 years prior to participating, or scored over the recommended threshold for their ages on the TICS™ (Telephone Interview for Cognitive Status™; Brandt & Folstein, 2003) within 2 weeks of participating in this study. Before starting the study, all participants did a red-green colour vision test. See Table 1 for participant demographics. No participants were excluded for being multivariate outliers at pre-test (using Mahalanobis distance value; Tabachnick et al., 2007). The PPLS Research Ethics committee approved this research and participants received £15 each for participating.

**Table 1.** Participant exclusions by age and strategy group.

Reason for exclusion	Younger adults		Older adults	
	Control	Strategy	Control	Strategy
Excluded from all analyses				
Cheating	1			
Non-compliant	—	6	—	11
Excluded from specific analyses				
Cheating		1^a^		
Missing data		2^b^		2^c^
Extreme outliers				
Multivariate outliers				
Colour vision^d^	1	1	1	2

RT: reaction time.

a One excluded from the training analysis.

b Post-test N-back digit (1), RTs in pre-test 2-back colours (1).

c Missing data in both N-back colours and RTs in pre-test 2-back letters (2).

d Colour-blind participants were excluded from colour N-back task.

### Procedure

**Figure 1.**
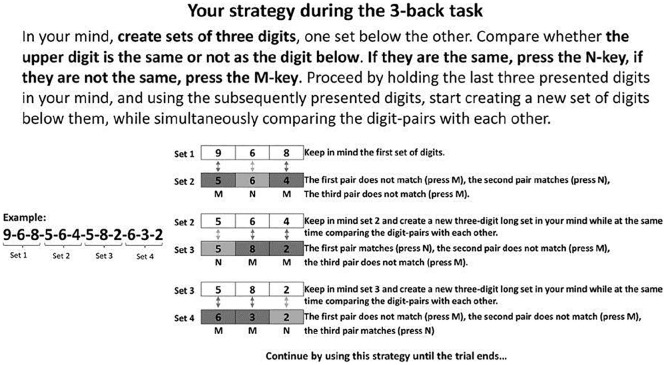
The visualisation strategy instructions for participants in the strategy groups during training.

We used a mixed pre- and post-test intervention design. First, participants completed a set of cognitive tasks (taking 1–1.5 hr) to assess baseline abilities. Two days later, they did a 30-min adaptive N-back task (*training session*). Half the participants from each age group were instructed to use a visualisation strategy (see Figure 1) during this training session (i.e., the strategy group), and the others performed the training without a strategy instruction (i.e., the control group). Two days later, participants completed the same set of cognitive tasks as on day 1. All participants were instructed to complete the pre-test session on a Monday, the training session 2 days later, and the post-test on Friday in the same week. They received instructions and an access link by email each night before the next session. At least 24 hr elapsed between sessions, and we did not exclude participants who completed sessions on slightly different days. Participants were not aware of the purpose of the study, nor that some were instructed to use a strategy and others not. They were instructed not to discuss study details with others who may also wish to take part. When they had completed the study, participants filled out a strategy questionnaire, reporting if they had used strategies and if so to describe those strategies. Participants were then informed about the purpose of the study, and the existence of the different groups.

Our procedure differed from that of Laine et al. (2018) as follows: (1) in contrast to Laine et al. (2018), we did not include a passive control group that did not perform any training between pre- and post-test, because the central question concerned the presence or absence of strategy instruction. (2) While all their participants were younger-adult university-level students, we also included a group of older adults. (3) Their participants performed pre- and post-test sessions in the laboratory while our participants completed all sessions online. (4) Our instructions and tasks were in English, theirs in Finnish. (5) We did not screen participants for health conditions (except for cognitive impairments in the older adults). Apart from these differences, our study was identical to theirs. We chose an online methodology because WM training software promoted by companies are typically intended for independent use with home computers or smartphones, and it enabled us to test a larger number of participants. However, there was a possibility of less attentive or compliant participants. To minimize the impact of this, we screened for outliers and asked participants if they used external tools (e.g., writing things down) when performing the tasks.

#### WM: training task

The strategy and control groups performed the same digit N-Back training task, but the strategy group was instructed to use the strategy illustrated in Figure 1. Participants saw digits (1–9) displayed one at a time, in the centre of the screen. They responded to each digit with the N or M key on their keyboard (meaning Yes or No, respectively) to indicate whether the current digit corresponded to the digit presented *n* items back in the sequence. After receiving task instructions, the actual training task started. Each sequence began with a blank screen (450 ms), followed by a digit (1,500 ms). Responses were recorded while the digit was on display or during the blank interval that followed. Hence, participants had a total of 1,950 ms to respond to each digit.

Each participant completed 20 blocks of 20 + *n* trials. All participants started at the 1-back level. However, the training was adaptive, so if 18–20 responses in a block were correct, *n* increased by one in the next block. If 15–17 responses were correct *n* remained the same, but following less than 15 correct responses, *n* decreased by one (or remained at one) in the next block. Each block contained randomised digit sequences with the constraint that each sequence included six targets (i.e., the digit was the same as the one displayed *n* digits back) and 14 non-targets. To prevent responses based on familiarity—enabling correct rejection based on not seeing that digit recently—four items out of the 14 non-targets were lures, that is, they were identical to a digit presented *n* ± 1 digits back (not applied to the 1-back condition). The maximum possible level was 9-back.

##### Strategy instruction

The strategy instruction taught participants to visualise the incoming *n* items as parallel digit strings (see Figure 1). For a 3-back sequence of 1-8-3-2-8-6, they would visualise 1-8-3 on top and 2-8-6 underneath. This strategy permitted visualised comparison of the upper and lower three digits, to judge whether they were identical. After comparing the two strings of digits the upper string would be discarded, and new digits were to be visualised as a new string, underneath. Participants in the strategy group were reminded of this strategy before each new block started.

##### Expectations

Prior to starting the training session, participants reported how much they thought they would improve on the training task during the session, using a 10-point Likert-type scale (1 = *No improvement at all*, 10 = *A large improvement*). Participants in the strategy group were informed about the strategy prior to giving these ratings, to capture differences in expectations associated with the instructed strategy. They also rated how much better they thought they would perform each of the tasks in the post-test session using a 1–10 Likert-type scale (1 = *The same performance as in the pre-test*, 10 = *A much better performance compared with the pre-test*).

##### Motivation and alertness

Before the training session, participants rated their motivation to perform the tasks and alertness on scales from 1 to 5.

#### Pre- and post-test measures

The following six cognitive tasks made up both the pre- and post-training test sessions and were thus completed by each participant twice, to compare performance improvement in participants who trained using the visualisation strategy with that observed in the control, no strategy group.

#### Criterion training task

##### Digit N-back

This was a shortened version of the adaptive training task described above, including 10 blocks instead of 20. Dependent variables were (1) the maximum digit level the participant had reached and (2) the average N-back level.

#### Untrained N-back tasks (task-specific near-transfer measures)

##### Letter N-back

This was a non-adaptive letter N-back task (2-back and 3-back), in which participants saw sequences of letters, and responded whether a given letter was identical to one presented 2 or 3 letters back. Participants did one block of the 2-back, one of the 3-back (order randomised) each containing 48 letters. Among these, 16 were targets, 32 non-targets, and half of the non-targets were lures (i.e., a letter identical to the letter presented next to the letter participants should base their response on; 8 *n* + 1 lures, 8 *n* − 1 lures). Each letter was shown for 1,500 ms, followed by a blank screen for 450 ms. Dependent variables: (1) accuracy (d-prime; Stanislaw & Todorov, 1999) and (2) mean reaction time (RT) on correct target responses.

##### Colour N-back

This was identical to the letter N-back task, but coloured squares were shown instead of letters.

#### Untrained WM tasks (task-general near-transfer measures)

##### Selective updating of digits

In this WM updating task (Murty et al., 2011), five digits between 1 and 9 were displayed on the screen in a row of five squares. Participants attempted to memorise the digit sequence. Then, a new row of five squares replaced the initial sequence. Two of the new squares contained digits, and three were empty. Participants were to replace the old digits with the new digits while maintaining the unchanged digits in memory. Each participant completed 10 trials with three such updating stages (i.e., new digits replaced original ones) and also 10 trials without updates. Participants saw the original five-digit sequence (4,000 ms), followed by a blank screen (100 ms), and the first updating stage (2,000 ms). At the end of each trial, participants reported the final five-digit sequence by clicking on the relevant digits in a recall grid with horizontally aligned squares containing numbers 1 to 9. All digit sequences followed these rules: (1) digit updates never occurred in adjacent squares, (2) adjacent digits deviated with more than one from each other (e.g., “2” could not be next to “1” or “3”), and (3) the two updated digits were never identical. Trial order was randomised between participants. The dependent variable was the percentage of correctly recalled digits (in the right order) in the updating trials.

##### Forward simple span

Participants were to remember sequentially presented digit sequences containing between 4 and 10 digits (one trial of each length) in order of appearance. Trial order was randomised for each participant. First, a fixation cross was shown in the middle of the screen (500 ms), followed by a digit (1,000 ms) and this procedure continued until all digits in the sequence had been presented. Then, participants recalled the digits by clicking on the correct digits (in the right order), displayed in horizontally aligned squares containing all possible digits (1–9). The dependent variables were (1) total number of correctly recalled digits in the correct serial position and (2) maximum span, that is, highest span length where all digits were recalled in the right order.

##### Running memory

Participants were instructed to report the final four digits of sequences containing between 4 and 11 items. A total of eight trials—one trial per sequence length—appeared in random order. First, a fixation cross appeared on the screen (500 ms), then a digit (1,000 ms), until the sequence ended. Participants then selected the final four digits in the same order as they had been presented, using a recall grid with horizontally aligned squares containing numbers 1–9. The dependent variable was the total number of correctly recalled items, in the correct position.

##### The strategy questionnaire

After completing all cognitive tasks in the post-training test session, participants filled out a questionnaire about their strategy use in each task they completed in the pre- and post-training test sessions, respectively. First, they responded to whether they had used a strategy (yes or no) for each specific task during the pre-test. If yes, they were asked to describe the strategy. They then indicated whether their strategy had changed between pre- and post-training tests (yes or no). If yes, they described their post-training test strategy.

## Results

### Exclusions

We excluded one younger adult in the control group who reported using pen and paper in the majority of the tasks. Also, one younger adult in the strategy group used pen and paper in one task and was excluded from that specific analysis. We excluded five participants with five or more errors on the Ishihara colour vision test from the colour N-back analyses and four participants from specific tasks due to missing data. See Table 1 for a summary of all exclusions by age and strategy group. Our results differed from Laine et al.’s (2018) in a way we had not anticipated—many of our strategy-group participants reported that they did not use the instructed strategy during training. In the original study, only 3 of 37 (8%) strategy-group participants failed to comply with the instruction, and non-compliant participants were not removed. In this study, 6 of 31 (19%) younger adults and 11 of 30 (37%) older adults in the strategy group reported not using the instructed strategy. We had not specified in our pre-registration how we would handle non-compliant participants. However, the aim was to replicate the study by Laine et al. (2018) with a different sample and test the effect of the instructed strategy in older adults. Hence, including non-compliant participants may lead to the trivial explanation that results did not replicate because too many of our participants did not use the strategy. Excluding non-compliant participants left 49 older and 54 younger adults, resulting in a power of .95 to detect the main effect on digit N-back performance observed by Laine et al. (2018) and a power of at least .80 to replicate the effects on untrained letter and colour N-back tasks. Therefore, we focused on results from compliant participants. For transparency, we present output from analyses including all participants in the supplementary materials and point out the differences. We also conducted exploratory analyses to confirm that non-compliant participants were not a less motivated or capable subset by comparing pre-test composite scores in younger and older compliant and non-compliant strategy participants (no significant differences; see Supplementary materials). We performed all analyses in the R environment version *3.5.1*, and the script and data are available via the OSF (https://osf.io/bwtuy).

### Background and pre-test characteristics

The control and compliant strategy groups did not differ significantly in years of education, gender distribution, or pre-test N-back composite performance in either age group (see Table 2). However, there was a significant age difference between control and strategy groups in older adults, such that participants in the strategy group were younger, *t*(47) = −2.53, *p* = 0.02. When non-compliant older adults in the strategy group were included, there were no age differences (see Supplementary materials), suggesting that the non-compliant older adults tended to be older.

**Table 2.** Demographics and pre-training N-back performance.

	Younger adults			Older adults		
	Control	Strategy	*p*	Control	Strategy	*p*
*N*	29	25		30	19	
Age	23.0 (3.96)	22.3 (3.22)	0.497	70.3 (5.69)	66.6 (3.82)	0.015
Gender F/M	21/8	19/6	1	20/10	12/7	1
Education	16.2 (2.81)	15.9 (2.68)	0.715	15.5 (3.43)	15.95 (2.5)	0.588
Pre-training N-back composite	0.28 (4.99)	–0.2 (5.62)	0.747	0.61 (4.88)	–1.4 (5.29)	0.197

Values in parentheses are standard deviations. *p*-values were calculated from *t*-tests for continuous variables and chi-square test for gender. The N-back composite scores were the summed values of the *z*-transformations of the average and maximum level accuracy in the adaptive digit N-back task, and d-prime values and Reaction Times for correct responses in the letter and colour N-back tasks.

### Alertness, motivation, and expectations

We assessed expected training-session improvement in participants in the strategy and control groups after the strategy participants had learned the strategy, but before starting the training. This was to check whether expectations were higher in the strategy groups, which might signal a placebo effect. There was no difference in expectations between control and strategy participants in younger, *t*(51) = 0.23, *p* = .82, or older adults, *t*(47) = 0.86, *p* = .39. Similarly, improvement expectations between pre-test and post-test did not differ for any of the tasks in either age group (all *p*-values ⩾.25). Self-reported alertness and motivation—assessed upon completion of the training session—also did not differ between strategy and control groups (all *p*-values ⩾.13). These measures were taken to test whether the strategy made the training more engaging. Similar results were observed when including non-compliant strategy participants (see Supplementary materials).

### Training session data

**Figure 2.**
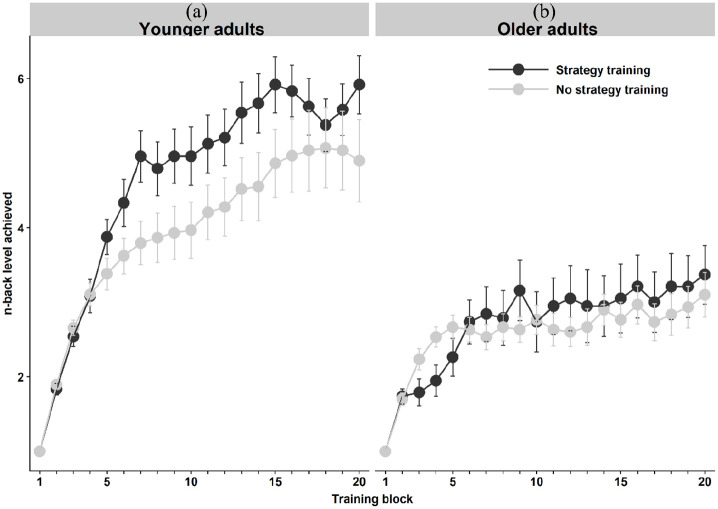
Performance across the 20 N-back digit training blocks, in the control and strategy groups in (a) younger and (b) older adults. Error bars represent standard errors of means.

Figure 2 shows performance over the 20 N-back blocks during the 30-min training session in the control and strategy groups in younger (panel a) and older adults (panel b). While Laine et al. (2018) found that participants using the instructed strategy outperformed control group participants already in the fourth training block, we found no differences in the fourth block in our younger adults, *t*(51) = −0.08, *p* = .94; controls *M* = 3.10 digits, strategy *M* = 3.08. However, among the older adults, the control group performed significantly better on the fourth N-back block than the strategy group, *t*(47) = −2.48, *p* = .02; controls *M* = 2.53 digits, strategy *M* = 1.93. To capture the curvilinear increases in performance across the 20 training blocks (see Figure 4), we performed an exploratory linear mixed-effects analysis using second-order orthogonal polynomials. The R packages lme4 (Bates et al., 2012) and lmerTest (Kuznetsova et al., 2015) were used in the model computation. Age Group, Strategy Group, and Block (coded both as a linear and a quadratic term) as well as all possible interactions were entered as fixed effects into the model. As random effects, we had participants’ individual intercepts. Visual inspection of residual plots did not reveal any obvious deviations from homoscedasticity or normality. Relations between performance levels in the two strategy groups across the training session did not differ between the age groups (Group × Age: Estimate = 0.14, *SE* = 0.13, *p* = .280). However, overall, strategy participants improved more across the training session than those training without a strategy, as evidenced in a Group × Block interaction both in the linear term (Estimate = −0.44, *SE* = 0.10, *p* < .001), and in the quadratic term (Estimate = 0.39, *SE* = 0.10, *p* < .001). Also, younger adults improved more across the training session than older adults, manifesting in a significant Block × Age interaction in the linear term (Estimate = −1.48, *SE* = 0.10, *p* < .001) as well as in the quadratic term (Estimate = 1.67, *SE* = 0.32, *p* < .001). There was no evidence for a three-way Group × Age × Block interaction in the linear term (Estimate = 0.05, *SE* = 0.10, *p* = .624). However, the quadratic term showed a statistically significant three-way interaction (Estimate = −0.24, *SE* = 0.10, *p* = .023), indicating that the relative effects of strategy across time differed between younger and older adults. Because the N-back training task was adaptive in its nature, with most of the participants managing the easiest levels, it is likely that only the quadratic term captured the increased learning rates among the younger strategy group, potentially stemming from increased demands on WM resources towards the end of the training session.

### The effects of training: pre- versus post-test performance

We tested whether training with the instructed strategy improved performance from pre- to post-training sessions on the various tasks to similar extents in the two age groups. Post-test performance was the dependent variable, pre-test performance the covariate, and strategy and age groups were between-subjects factors. See Tables 3 and 4 for pre- and post-training descriptives (means, standard deviations, pre-post correlations, and effect sizes) for each group, and Table 5 for analysis of covariance (ANCOVA) statistics. To adjust for multiple comparisons, we applied Benjamini-adjusted *p*-values for group comparisons on each pre-post outcome measure (Benjamini & Hochberg, 1995).

**Table 3.** Mean values (standard deviations) for the pre-post measures per group at pre- and post-test, for younger adults.

	Control group (*N* = 29)				Strategy group (*N* = 25)			
	Pre	Post	*r*	*d*	Pre	Post	*r*	*d*
Trained digit N-back								
Maximum level	4.28 (1.71)	5.52 (2.16)	0.66	0.71	4.04 (1.49)	6.75 (1.59)	0.32	1.75
Average level	2.72 (0.91)	3.41 (1.02)	0.70	0.62	2.67 (0.94)	4.13 (0.81)	0.35	1.66
Untrained N-back tasks (task-specific near transfer)								
Letter 2-back (d-prime)	2.25 (0.94)	2.48 (0.96)	0.71	0.24	2.19 (1.05)	3.01 (0.85)	0.38	0.85
Letter 3-back (d-prime)	1.19 (0.76)	2.00 (1.15)	0.55	0.80	1.12 (1.10)	2.67 (0.91)	0.40	1.53
Colour 2-back (d-prime)	2.03 (0.78)	2.54 (0.93)	0.39	0.59	2.14 (1.08)	2.85 (1.03)	0.62	0.68
Colour 3-back (d-prime)	0.90 (0.82)	1.69 (1.22)	0.52	0.74	1.03 (0.59)	2.53 (0.96)	0.47	1.79
Letter 2-back RT (ms)	803.85 (108.04)	686.08 (127.44)	0.51	–0.99	784.43 (127.91)	636.02 (151.96)	0.40	–1.05
Letter 3-back RT (ms)	802.58 (120.04)	676.99 (100.28)	0.36	–1.13	787.27 (206.49)	623.85 (132.05)	0.38	–0.92
Colour 2-back RT (ms)	811.47 (119.21)	696.87 (115.34)	0.26	–0.98	811.88 (124.40)	661.41 (146.82)	0.29	–1.10
Colour 3-back RT (ms)	857.86 (129.50)	721.56 (106.42)	0.24	–1.15	817.49 (225.25)	661.63 (150.60)	0.20	–0.81
Other untrained WM tasks (task-general near transfer)								
Selective updating of digits	32.38 (8.14)	33.00 (7.08)	0.78	0.08	35.32 (8.53)	37.24 (7.60)	0.69	0.24
Digit span (correct items)	34.52 (10.00)	34.10 (8.83)	0.73	–0.04	35.16 (9.33)	37.76 (7.47)	0.22	0.31
Digit span (maximum span)	6.79 (2.06)	7.28 (1.53)	0.70	0.25	7.36 (2.00)	7.88 (1.54)	0.26	0.29
Running memory	25.31 (4.49)	26.28 (5.32)	0.49	0.19	24.92 (4.97)	27.20 (4.38)	0.53	0.48

RT: reaction time; WM: working memory.

Values in parentheses are standard deviations. *r* = correlation between pre- and post-test. Cohen’s *d* represents effect sizes for correlated samples. Exclusions to specific analyses apply.

**Table 4.** Mean values (standard deviations) for the pre-post measures per group at pre- and post-test, for older adults.

	Control group (*N* = 30)				Strategy group (*N* = 19)			
	Pre	Post	*r*	*d*	Pre	Post	*r*	*d*
Trained digit N-back								
Maximum level	3.10 (0.92)	3.83 (1.29)	0.51	0.64	2.79 (0.71)	3.95 (1.35)	0.39	1.02
Average level	1.94 (0.55)	2.55 (0.76)	0.58	0.89	1.96 (0.45)	2.56 (0.79)	0.50	0.87
Untrained N-back tasks (task-specific near transfer)								
Letter 2-back (d-prime)	1.85 (0.79)	2.31 (0.86)	0.63	0.55	1.84 (0.72)	2.19 (0.86)	0.40	0.43
Letter 3-back (d-prime)	0.76 (0.48)	1.28 (0.88)	0.45	0.68	0.80 (0.65)	1.45 (1.01)	0.43	0.74
Colour 2-back (d-prime)	1.81 (0.75)	2.09 (0.83)	0.53	0.35	1.36 (0.86)	2.08 (1.01)	0.32	0.76
Colour 3-back (d-prime)	0.77 (0.58)	0.94 (0.76)	0.16	0.24	0.44 (0.46)	0.94 (0.71)	0.25	0.82
Letter 2-back RT (ms)	1,017.30 (165.77)	869.92 (178.94)	0.82	–0.85	1,021.58 (177.47)	915.14 (145.94)	0.49	–0.65
Letter 3-back RT (ms)	1,002.24 (174.61)	936.59 (167.59)	0.70	–0.38	983.38 (158.56)	922.80 (151.06)	0.46	–0.39
Colour 2-back RT (ms)	1,013.51 (166.92)	909.24 (160.31)	0.64	–0.64	1,050.99 (168.84)	951.99 (128.94)	0.84	–0.60
Colour 3-back RT (ms)	1,071.56 (160.84)	959.52 (199.16)	0.54	–0.61	1,010.70 (176.35)	1,026.44 (158.01)	0.45	0.09
Other untrained WM tasks (task-general near transfer)								
Selective updating of digits	24.63 (11.48)	30.43 (11.33)	0.75	0.51	25.79 (11.59)	29.53 (10.50)	0.75	0.34
Digit span (correct items)	33.23 (8.24)	34.37 (7.91)	0.64	0.14	32.37 (8.54)	32.74 (7.78)	0.74	0.04
Digit span (maximum span)	6.93 (1.36)	7.23 (1.36)	0.18	0.22	6.79 (1.65)	6.74 (1.63)	0.64	–0.03
Running memory	24.33 (4.33)	23.80 (5.29)	0.51	–0.11	23.32 (5.63)	24.37 (4.19)	0.59	0.21

RT: reaction time; WM: working memory.

Values in parentheses are standard deviations. *r* = correlation between pre- and post-test. Cohen’s *d* represents effect sizes for correlated samples. Exclusions to specific analyses applied.

**Table 5.** ANCOVA results for the trained task and for the transfer measures.

		*F*	*p*	$d/\eta_{\text{p}}^{\text{2}}$
Trained digit N-back				
Maximum level	**Strategy**	**8.73**	**.008**	**0.61**
	**Age**	**20.57**	**<.001**	**0.88**
	Interaction	3.45	.066	0.034
Average level	**Strategy**	**6.53**	**.015**	**0.53**
	**Age**	**21.25**	**<.001**	**0.87**
	**Interaction**	**6.69**	**.015**	**0.064**
Untrained N-back tasks (task-specific near transfer)				
Letter 2-back (d-prime)	Strategy	2.27	.204	0.33
	Age	3.76	.111	0.32
	Interaction	5.21	.066	0.050
Letter 3-back (d-prime)	Strategy	5.75	.055	0.50
	**Age**	**16.85**	**<.001**	**0.78**
	Interaction	2.40	.204	0.024
Colour 2-back (d-prime)	Strategy	1.95	.235	0.29
	Age	3.95	.109	0.42
	Interaction	0.01	.924	<.001
Colour 3-back (d-prime)	**Strategy**	**6.96**	**.033**	**0.57**
	**Age**	**25.98**	**<.001**	**1.00**
	Interaction	2.26	.204	0.024
Letter 2-back (RT in ms)	Strategy	0.01	.924	<.001
	Age	8.11	.021	–0.57
	Interaction	2.64	.198	0.026
Letter 3-back (RT in ms)	Strategy	1.17	.356	–0.23
	**Age**	**48.32**	**<.001**	**–1.47**
	Interaction	0.71	.483	0.007
Colour 2-back (RT in ms)	Strategy	0.06	.889	–0.07
	**Age**	**20.43**	**<.001**	**–0.98**
	Interaction	1.44	.312	0.015
Colour 3-back (RT in ms)	Strategy	0.42	.59	0.09
	**Age**	**44.02**	**<.001**	**–1.36**
	Interaction	4.78	.075	0.049
Other untrained WM tasks (task-general near transfer)				
Selective updating of digits	Strategy	0.04	.987	0.06
	Age	0.38	.715	–0.18
	Interaction	2.47	.309	0.025
Digit span (correct items)	Strategy	0.67	.624	0.19
	Age	1.00	.55	0.14
	Interaction	2.94	.309	0.029
Digit span (maximum span)	Strategy	0.01	.987	<.001
	Age	3.58	.309	0.33
	Interaction	2.35	.309	0.023
Running memory	Strategy	1.72	.385	0.26
	Age	5.34	.276	0.47
	Interaction	<.001	.987	<.001

RT: reaction time; WM: working memory.

To adjust for multiple comparisons, Benjamini–Hochberg adjusted *p*-values were applied for group comparisons on each pre-post outcome measure. Cohen’s *d* is presented for the group comparisons, $\eta_{\text{p}}^{\text{2}}$ for the interactions. Significant factors are presented in bold font.

#### The trained N-back task with digits

**Figure 3.**
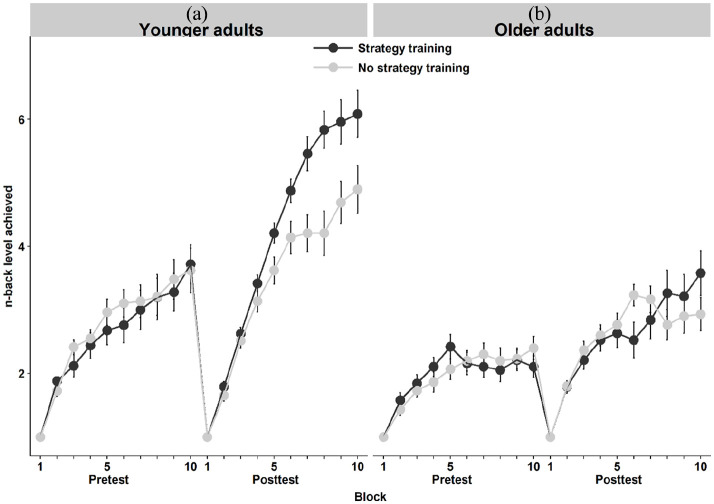
Average performance across the 10 blocks of the trained N-back task at pre- and post-test in the control and strategy groups in (a) younger and (b) older adults. Error bars represent standard errors of means.

A 2 (Group) × 2 (Age) between-subjects ANCOVA of maximum post-test N-back performance that controlled maximum pre-test N-back performance indicated significant main effects of strategy, *F*(4, 97) = 8.73, *p* = .008, *d* = 0.61, and age group, *F*(4, 97) = 20.57, *p* < .001, *d* = 0.88, but no significant interaction, *F*(4, 97) = 3.45, *p* = .066, $\eta_{\text{p}}^{\text{2}}=.03$ . For average digit N-back performance, there were also significant effects of strategy, *F*(4, 97) = 6.53, *p* = .015, *d* = 0.53, and age group, *F*(4, 97) = 21.25, *p* < .001, *d* = 0.87, as well as a significant interaction, *F*(4, 97) = 6.69, *p* = .015, $\eta_{\text{p}}^{\text{2}}=.06$ . The strategy manipulation appeared more beneficial in younger adults (see Figure 3). When including non-compliant participants, no effect of strategy group was observed for maximum digit level in either age group; however, there was a significant interaction between age group and strategy level for average digit N-back performance (see Supplementary materials).

As an additional exploratory analysis, we also examined the block-level improvement in the trained digit N-back task at post-test using a linear mixed-effects analysis. As in the training analysis (see section “Training session data”), the fixed effects consisted of Age Group, Strategy Group, and Block (coded both as a linear and a quadratic term) together with their interaction terms. Moreover, we included the maximum reached digit N-back level at pre-test as a time-invariant covariate to control for possible group differences prior to intervention. Participant served as the random effect. The results showed a significant Group × Age interaction (Estimate = 0.17, *SE* = 0.07, *p* = .018), indicating that the instructed strategy was more effective for the younger adults. The linear interaction term of Block × Group was statistically significant (Estimate = −0.15, *SE* = 0.08, *p* < .001), whereas the corresponding interaction in the quadratic term was not (Estimate = −0.15, *SE* = 0.08, *p* = .065). Similarly, a significant Block × Age interaction was observed in the linear term (Estimate = −1.34, *SE* = 0.08, *p* < .001), but not in the quadratic term (Estimate = 0.15, *SE* = 0.08, *p* = .067). We observed a statistically significant three-way Group × Age × Block interaction both in the linear term (Estimate = 0.27, *SE* = 0.08, *p* = .001) and in the quadratic term (Estimate = −0.23, *SE* = 0.08, *p* = .006), indicating that the younger adults benefitted more of the instructed strategy across the blocks as compared to the older adults.

### Untrained N-back tasks (task-specific near-transfer)

#### Letter N-back

There was no significant effect of age or strategy group on d-prime in the Letter 2-back, and no interaction (all *p*’s ⩾ .066). There was no significant main effect of strategy in the more demanding 3-back condition, *F*(4, 98) = 5.75, *p* *=* .055, *d* = 0.50, despite a medium effect size. This was the only instance where our results regarding the strategy manipulation deviated from Laine et al.’s (2018). We observed a statistically significant main effect of age group, *F*(4, 98) = 16.85, *p* < .001, *d* = 0.78, but our Strategy × Age interaction was non-significant, *F*(4, 98) *=* 2.40, *p =* .204, *$\eta_{\text{p}}^{\text{2}}=.02$ *. There were significant effects of age on RTs in both the 2-back and the 3-back tasks, but no effects of strategy group, nor any interactions between strategy and age group (see Table 5). Results were similar when including non-compliant participants (see Supplementary materials).

#### Colour N-back

We excluded five participants with five or more errors on the Ishihara colour vision test from these analyses. There was no significant main effect of strategy group for the 2-back d-prime (*p* = .29), but strategy group showed more improvement on the more demanding 3-back task, *F*(4, 93) = 6.96, *p* = .033, *d* = 0.57. Correspondingly, we observed a significant main effect of age in the 3-back, *F*(4, 93) = 25.98, *p* < .001, *d* = 1.00, but not the 2-back task, *F*(4, 93) = 3.95, *p* = .109, *d* = 0.42. There were no interactions between age and strategy (both *p*’s ⩾.204). The older adults were significantly slower in both the 2-back and 3-back tasks, but there were no effects of strategy group, nor any interactions between strategy and age group (see Table 5). When including non-compliant participants results were similar, but no effect of strategy group in the 3-back task was observed (see Supplementary materials).

### Other untrained WM tasks (task-general near-transfer)

There were no significant main effects either of age or strategy group nor any interactions for selective updating of digits, running memory, or either forward digit span measure (correctly recalled digits, or maximum span), all *p*’s ⩾ .276. The same pattern of results was found including non-compliant participants (see Supplementary materials).

### Self-generated strategies and performance

**Figure 4.**
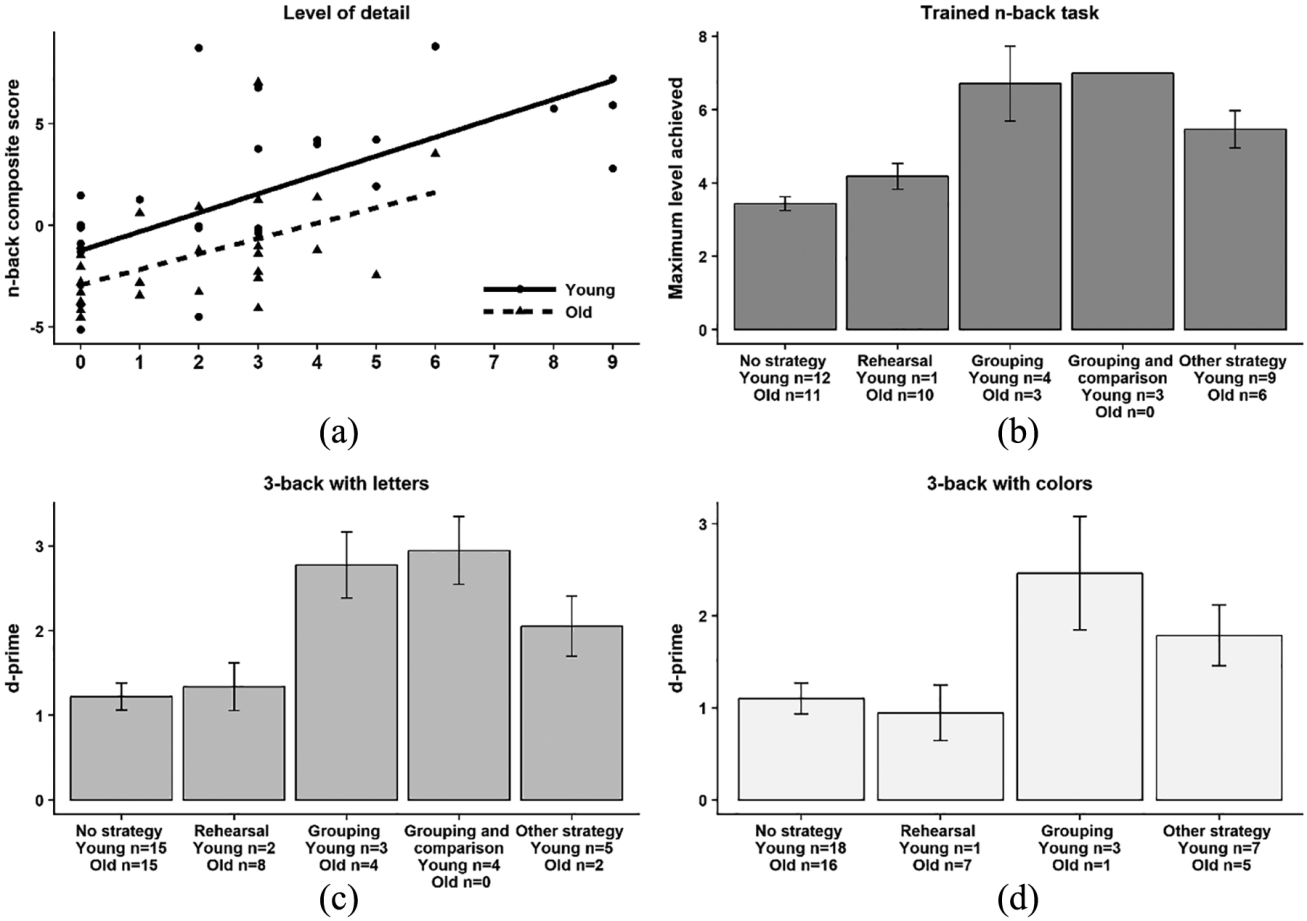
(a) Regression plot with level of detail of reported strategies (9 = maximum level of detail) as the independent variable (X-axis) and the N-back composite score (Y-axis) as the dependent. The N-back composite summed up post-test *z*-values of average and maximum N-back level reached in the trained digit N-back task, and the d-prime values in the untrained letter and colour 3-back tasks. (b) Strategy type and performance in the trained N-back digit task at post-test. (c) Strategy type and performance in the untrained letter N-back task at post-test. (d) Strategy type and performance in the untrained colour N-back task at post-test. Whiskers in panels (b)–(d) represent standard errors of means. The three participants using Grouping and Comparison in the Trained N-Back task all reached the same level, hence no error bar.

We tested whether (1) the types of reported self-generated strategies and (2) the reported levels of detail of those strategies were associated with better post-test N-back performance in control group participants. Only control participants were used to obtain a “pure” measure of spontaneously generated strategies in participants who were not exposed to any strategy instruction. One older adult was excluded due to missing strategy data for N-back letters and colours. Thus, the final sample of controls included 58 participants. The types of strategies and level of detail reported in the two age groups are presented in Figure 4.

#### Self-generated strategies: type

We classified self-generated post-test strategies according to Laine et al.’s (2018) classification scheme, based on categories used by Morrison et al. (2016). Two independent raters classified each strategy report into one of these categories: Rehearsal, Grouping, Updating, Grouping and Comparison, Semantics, Phonology, Imagery, Familiarity, Guessing, Other Strategy, or No Strategy (see Supplementary Table S5). Initial inter-rater reliability (unweighted Cohen’s kappa) for the three N-back tasks was consistent and good: trained digit N-back (κ = .79, 95% confidence interval, CI = [0.72, 0.86]), letter N-back (κ = .81, 95% CI = [0.74, 0.88]), and colour N-back (κ = .81, 95% CI = [0.73, 0.88]). The raters then resolved discrepancies through discussion consensus, producing the final strategy type classifications used in the analysis. Strategies reported by less than 5% of participants were grouped as “Other Strategy” (see Supplementary Table S6 for the distributions of strategy types used in the three N-back tasks at post-test). The final list comprised five categories for the digit and letter N-back (No Strategy, Rehearsal, Grouping, Grouping and Comparison, and Other Strategy) and four categories for the colour N-back (No Strategy, Rehearsal, Grouping, and Other Strategy). We tested if N-back performance differed by strategy type using one-way analyses of variance (ANOVAs). No strategy served as the baseline. In each model, the dependent variable was N-back post-test performance and strategy type was the between-subjects factor. Figure 4 shows N-back post-test performance as a function of strategy type at post-test for each N-back task. We did not include age as a factor given the limited number of observations but, see Figure 4 for usage by age group.

##### Digit N-back (maximum level)

Reported strategy use was associated with significantly better performance than not using a strategy, *F*(4, 54) = 9.75, *p* < .001, $\eta_{\text{p}}^{\text{2}}=.42$ . Participants in the No Strategy group were outperformed by participants who reported using Grouping, *t*(28) = 4.49, *p* < .001, *d* = −2.22, and Grouping and Comparison, *t*(24) = 2.39, *p* = .02, *d* = −4.16. However, those not reporting using strategies did not differ in performance from those using Rehearsal, *t*(32) = 1.32, *p* = .192, *d* = −0.76, or Other Strategy, *t*(36) = 0.27, *p* = .79, *d* = −1.44.

##### Letter 3-back (d-prime)

Using a strategy was significantly better than not using a strategy, *F*(4, 53) = 7.17, *p* < .001, $\eta_{\text{p}}^{\text{2}}=.35$ . Again, participants in the No Strategy group were outperformed by participants using Grouping, *t*(35) = 3.96, *p* < .001, *d* = −1.72, and Grouping and Comparison, *t*(32) = 2.45, *p* = .018, *d* = −1.99, but not by those using Rehearsal, *t*(38) = 0.36, *p* = .721, *d* = −0.13, or Other Strategy, *t*(35) = −0.05, *p* = .964, *d* = −0.94.

##### Colour 3-back (d-prime)

Again, using a strategy was better than not using a strategy, *F*(3, 54) = 3.39, *p* = .025, $\eta_{\text{p}}^{\text{2}}=.16$ . Participants using Grouping performed significantly better than those using No Strategy, *t*(36) = 2.61, *p* = .012, *d* = −1.37. There was no difference between No Strategy and Rehearsal *t*(40) = −0.35, *p* = .729, *d* = 0.16, or between No Strategy and Other Strategy, *t*(44) = 0.77, *p* = .444, *d* = −0.67.

##### Verbal rehearsal in older adults: exploratory analyses

Perhaps Rehearsal was not associated with better performance compared to No Strategy because Rehearsal was primarily used by older adults, who may generally perform worse than younger adults. To test this possibility, we performed exploratory analyses comparing older adults using Rehearsal with older adults using No Strategy, for the three different N-back tasks.^2^ For the letter N-back (3-back d-prime), there were no differences, *t*(21) = 0.11, *p* = .92, *d* = −0.05, nor for the colour N-back (3-back d-prime), *t*(20) = −0.98, *p* = .34, *d* = −0.47. However, for the digit N-back (maximum level), Rehearsal was associated with better performance than No Strategy, *t*(19) = −2.21, *p* = .04, *d* = −0.96.

#### Self-generated strategies: level of detail

We tested whether the level of detail of the reported strategy during post-test was associated with post-test N-back performance in controls. The same raters as above scored the reported strategies based on the criteria used by Laine et al. (2018) on a scale from 0 to 3. Zero meant that participants did not report using a strategy. One point was given to a vague, non-specific strategy (e.g., “I memorised the digits in my mind”) and two points for a clear strategy with at most one detail (“I memorised the digits in pairs, such as 52–48”). Scorers gave three points for clearly described strategies with at least two details (e.g., “I split the digits into different series, and compared those to each other”). The raters scored the three N-back varieties (digit, letter, and colour), such that each participant had a total N-back level-of-detail score between 0 and 9.

There was good interrater reliability between the two independent raters for this scoring procedure (linearly weighted kappa analysis; κ_w_ = .83, 95% CI = [0.80, 0.86]; Cohen, 1968). The raters then discussed and reached consensus on all discrepant scores, producing a final level of detail score for each control group participant. To test if these scores predicted general N-back post-test performance, we calculated an N-back composite score including: (1) for the trained digit N-back task: summed values of the z-transformations of the post-test average and maximum level reached, and (2) post-test d-prime variables in the letter and colour 3-back tasks.

We performed a multiple regression analysis with the N-Back composite score serving as the dependent variable, and level of strategy detail and age group serving as predictors. The results showed a significant regression equation, *F*(3, 52) = 18.15, *p* < .001, with an adjusted *R*^2^ of 0.48. Level of detail was significantly associated with post-test N-back composite performance (β = 0.564, *t* = 4.99, *p* < .001), whereas age group was not (β = −0.285, *t* = −1.66, *p* = .104), and there was no evidence for an interaction (β = −0.057, *t* = −0.51, *p* = .614; see Figure 4, panel a).

## Discussion

This study tested the Strategy Mediation Hypothesis of WM training via external (i.e., instructed) and internal (i.e., spontaneously self-generated) strategy use in a single session of adaptive N-back training. It was a systematic replication of the study by Laine et al. (2018) to test the validity of their results for younger adults in a different sample of participants (see Simons, 2014). We also explored potential implications of strategy use in N-back training in healthy older adults, given that they are often targeted by commercial training programmes (e.g., Federal Trade Commission, 2016).

The instructed N-back strategy was associated with greater performance improvement during the training session across the 20 training blocks in younger adults and was associated with significantly better performance on the trained N-back digit task a few days later, during the post-test session. However, the older adults appeared to benefit less from strategy instruction across blocks than the younger adult strategy group (see Figure 2). Instructed strategy was also associated with significantly more accurate performance on the more difficult version of the untrained colour N-back task (3-back) in both age groups, without improved reaction times—similar to transfer patterns typically seen after weeks of ordinary adaptive WM training (Soveri et al., 2017), and similar to Laine et al.’s (2018) observations. However, even though the effect size of the strategy (i.e., Control group vs Strategy group) was moderate following training in the untrained letter N-back (*d* = 0.50), after correcting for multiple comparisons, we did not replicate the beneficial effect of strategy on the untrained letter N-back. This is difficult to interpret. Perhaps including older adults, who appeared to struggle with implementing the strategy—especially across earlier blocks—for the digit N-back tasks increased variability in our ANCOVA models. As expected, there was no effect of strategy group on any of the structurally different WM tasks (i.e., no task-general near transfer). These tasks tested memory for digits—like the trained task—but did not require comparison, making the instructed strategy inapplicable. No effects of strategy group on these structurally different WM tasks indicated that improved performance was not driven by increased motivation or paying more attention to the tasks, nor to thinking more about how to perform well. It also indicated that even though performance increased on the N-back tasks (mainly in younger adults, as evidenced in our exploratory block-level analysis on digit N-back post-test performance), there appeared to be no improvement in WM ability (which would arguably be unexpected following such a short training session).

These results indicate that learning to use a specific strategy—which is unlikely to improve general reasoning ability or prevent age-related cognitive decline—can produce significant N-back performance gains. This has several implications for the training literature. First, our results were in line with the notion that much of N-back training is task-specific (Soveri et al., 2017). Before encouraging members of the public to spend time and money on cognitive training, it should be established that improvements are not limited to some task-specific strategic approach—which is probably nearly useless in the individuals’ lives. Some training programmes keep users engaged via task-improvement feedback, suggesting that better performance implies improved WM ability. However, our findings of significant strategy-induced task-specific near transfer without task-general near transfer, along with those from many other studies, suggest that such claims are vastly overstated.

Strategy-induced improvements raise further questions regarding whether training strategies can be applied to outcome variables claimed to reflect far transfer. If so, perhaps some types of training are associated with far transfer improvement because trained participants develop a strategy which generalises to the outcome measure. Further research should explore whether strategies developed during training are applied to seemingly unrelated outcome measures. For instance, tests assumed to measure “fluid” intelligence (e.g., Raven’s Matrices) are often used as measures of far-transfer training gains. Cogmed’s visual processing and matching training is similar to Raven’s Matrices (Shipstead et al., 2012). Using a speeded-up version of Raven’s Matrices (e.g., Jaeggi et al., 2008) may even increase these similarities (Chuderski, 2013). Moreover, some evidence suggests that opportunity to practice may improve performance on Raven’s Progressive Matrices (e.g., Blieszner et al., 1981; Denney & Heidrich, 1990; Klauer et al., 2002). Thus, training control groups on a different task can be misleading if it differs in terms of structural similarity from outcome measures. If a WM training paradigm only improves performance on one specific reasoning measure, strategy mediation in far-transfer measures needs to be ruled out. Arguably, transfer should generalise to several structurally different outcome tasks, before transfer to for instance “fluid” intelligence is asserted.

However, evidence that strategy use improves performance on trained tasks does not falsify the Capacity Hypothesis of WM training; it is still possible that training also usefully improves cognitive capacity. According to the Capacity Hypothesis, training works by challenging the cognitive system, and working at one’s capacity limits is considered a prerequisite for the sorts of plastic changes in the brain considered to reflect increased capacity (e.g., see Klingberg, 2010). If strategies reduce cognitive load by making the task easier, this might prevent capacity-increasing change and therefore prevent broader transfer. Strategy use may, therefore, produce problematic confounds in training studies either by making possible improvements without meaningfully increasing cognitive capacity or by preventing optimally “broad,” efficient training.

The assumption that online cognition is limited by the capacity of a domain-general attentional resource or WM system (Engle & Kane, 2004) which can be “trained” and thus improve cognitive abilities more broadly (Jaeggi et al., 2008) underlies the Capacity Hypothesis. The finding that a visualisation strategy was associated with improved memory performance might fit better with theories of WM as containing a variety of cognitive systems among which participants may choose according to task demands (Baddeley & Logie, 1999; Logie, 2011; Logie & Niven, 2012). Encouraging participants to use other sub-components of the cognitive system (e.g., visualising the strings of digits) appeared to boost performance significantly, as suggested by Logie (2012). Strategic “off-loading” from a general resource to another system might be useful by freeing up its cognitive resources (McNamara & Scott, 2001). This would not imply that a general resource cannot be trained at all, but it suggests that this resource was not necessarily trained as was assumed in many training studies.

While our results suggest that instructed strategies can play a significant role in WM performance, strategies arguably only have implications for the training literature if participants spontaneously use them during adaptive training (e.g., Dunning & Holmes, 2014), which needs to be demonstrated. Our results from the non-instructed group suggested that participants did generate and use strategies spontaneously. Both strategy type and level of detail (i.e., how elaborate the strategy was) were associated with higher performance on all three N-back tasks at post-test (see Figure 4). However, the categories used in our study did not capture all strategies (16.1% classified as “Other” across the three tasks). Strategies classified as “Other” were not associated with improved performance in either N-back tasks (compared to not using a strategy). This suggests that a substantial proportion of participants applied potentially inefficient strategies. The implications of such strategies for the training literature are unclear, and more detailed research into the causes—and consequences—of these “Other” self-generated strategies may help design better training paradigms.

Moreover, the beneficial effects of spontaneous self-reported strategies on performance may be inflated. For instance, strategies may be used more by high-capacity individuals, who have more cognitive resources available for generating effective strategies while performing the task (Dunlosky & Kane, 2007) and who may also be more likely to reap training benefits regardless of strategy use. As well, reports of strategy use could be influenced by general task motivation, if participants who tried their best on the task are also keener to provide detailed descriptions of their approaches. Therefore, explicitly manipulating strategy use via instructed strategies that participants can and do use is important to ensure that associations between performance and strategies are not driven by such confounds. Our instructed strategy manipulation suggested that most participants can benefit from using a strategy—but an unexpected limitation was the relatively large proportion of non-compliant participants, whom we excluded from the main analyses. While WM capacity appeared similar in compliant and non-compliant participants (indicated by no significant differences in pre-test composite scores), we cannot infer whether non-compliant participants were unable to apply the strategy or preferred not to. However, despite these limitations regarding the causes of whether or not a strategy is applied, our results suggest that both internally generated and externally instructed strategies can boost N-back performance. The brevity of the training session (30 min) limits the generalisability of our findings to the broader training literature, where training is typically conducted over several weeks (e.g., von Bastian & Oberauer, 2014). Another limitation of the design is that we cannot infer whether the instructed strategy improved performance because participants used it during the training session, or simply because they were exposed to it. A third group of participants who trained without a strategy, and then learnt about the strategy just after the training session, would be needed to test this. From the data we do have, it seems that younger adults in the strategy group started benefitting immediately (see Figure 2), suggesting that this specific strategy in the N-back task did not require extended practice but may be implemented right away. Nonetheless, perhaps in older adults more training with the strategy would have made it more beneficial. However, a recent study investigated the effect of the same instructed strategy during a 4-week training period, in younger adults (Fellman et al., 2020). While the beneficial effect of strategy training replicated, their results indicated that the beneficial effect of the N-back strategy was short-lived, mostly visible during the first training session. Fellman et al. speculated that the instructed training may tie the hands of the trainees too much, while the uninstructed trainees were free to develop and optimise their own strategies. It is unclear whether older adults would have been able to benefit more if exposed to such extended strategy training.

### Strategy training in healthy older adults

We included healthy older-adult participants to compare their strategy use with that of younger adults, noting both similarities and differences. During training, the older adult strategy group appeared to benefit less from training than the younger-adult strategy group (see Figure 2). In the post-test, younger and older adults both benefitted from the strategy in the untrained N-back colour 3-back, and in the maximum digit N-back score. However, in the average digit N-back level attained, the older adults benefitted less, reflecting that, on average, the control group outperformed the strategy group until block 8 of 10 (see Figure 3).

Some previous studies instructing participants to apply mnemonic techniques or strategies have found more substantial training gains in younger than in older adults (e.g., Lövdén et al., 2012; Verhaeghen et al., 1992; Verhaeghen & Marcoen, 1996 but see Gross et al., 2012). Taken together, our results suggested that while both age groups at least partially benefitted from the strategy, older adults appeared to benefit more slowly, as implementing the new strategy reduced performance during early trials. If participants develop spontaneous strategies during uninstructed, regular training and younger participants generate and effectively apply them more quickly, our results might be consistent with observations of initially larger gains in younger adults, followed by comparable improvements in both age groups in the final weeks (e.g., Brehmer et al., 2012). Furthermore, a large proportion of our older adults (11 of 30) did not use the instructed strategy, possibly indicating that they found it difficult to implement. Perhaps if implementing a strategy is generally more challenging for older than younger adults, it is also more beneficial once they learn how to do it effectively. For instance, cognitive training using an episodic memory strategy task was associated with less age-related decline in white matter microstructures in healthy older adults compared to a control group, after 40 weeks (de Lange et al., 2017).

Also, it is possible that older adults struggled to implement the strategy because it was visually based—some previous research suggests that visual WM declines more in healthy ageing than verbal WM (e.g., Johnson et al., 2010). Similarly, more older than younger adults in our uninstructed control group reported using a sub-vocal Rehearsal strategy, that is, silent repetition of verbal labels for material to be recalled (see Logie et al., 1996; Wang et al., 2016). Specifically, 4 younger and 25 older adults used this strategy in the three N-back tasks combined (see Figure 4), supporting previous suggestions that older adults may rely more on verbal rehearsal even in visual WM tasks (Forsberg et al., 2019). More severe WM deficits for visuospatial material than for verbal material have been observed in older adults (e.g., Jenkins et al., 1999; Leonards et al., 2002; Myerson et al., 1999), and perhaps sub-vocal rehearsal can be used to compensate for declining visual memory. Rehearsal benefitted older adults in our digit N-back task (compared to those not using a strategy), in line with observations that older adults’ WM benefitted from verbal encoding strategies (Bailey et al., 2014). However, it was not beneficial in the letter or colour N-back tasks. Verbal rehearsal might have been most useful for the digit task because the letter set likely produced more phonological similarity effects (Salamé & Baddeley, 1986), and colour names are longer, thus less efficient to rehearse (Schweickert et al., 1990). Also, the digit N-back task was adaptive (maximum levels reached by older adults: control group *M* = 3.83, *SD* = 1.29; strategy group *M* = 3.95, *SD* = 1.35)—in contrast to the letter and colour tasks, which only tested accuracy at 2- and 3-back levels. One can only speculate whether rehearsal benefits on accuracy might have been evident in these tasks when moving beyond 2- and 3-back.

In the broader training literature, younger adults often improve more than older adults (Bürki et al., 2014; Heinzel et al., 2014; Li et al., 2008; Zinke et al., 2014)—but gains of similar magnitude on trained tasks in younger and older adults are also sometimes observed (e.g., Bürki et al., 2014; Li et al., 2008; Richmond et al., 2011; von Bastian et al., 2013; Zając-Lamparska & Trempała, 2016). However, training of executive functions appeared to yield greater training-related benefits in older than in younger adults (e.g., see Karbach & Kray, 2016; Kray & Lindenberger, 2000). Larger training gains in younger adults are thought to be consistent with animal models suggesting that older age is associated with less neuroplastic change (Blumenfeld-Katzir et al., 2011; van Praag et al., 2005). Our results suggest an alternative explanation: perhaps younger adults appear to benefit more from training because they are more adept at developing strategies. Furthermore, age differences in training gains between paradigms may be driven by differences in strategy effectiveness (e.g., visual vs verbal). The observed age differences in the effectiveness of the instructed visualisation strategy and the use of spontaneous verbal rehearsal strategies fit with literature suggesting that not all cognitive functions decline with age to the same degree (for reviews, see Logie & Morris, 2015; Perfect & Maylor, 2000). In sum, these results support the notion that overall N-back performance may reflect use of different cognitive resources in different participants (Johnson et al., 2010; Logie, 2018; Thurstone, 1931).

To conclude, our results supported Laine et al.’s (2018) conclusion that using a visualisation strategy during training improved N-back performance in younger adults. Furthermore, the strategy also at least partly improved performance in older adults. The results provided support for the Strategy Mediation hypothesis of training and suggest that strategies can enable more efficient use of a limited WM capacity, which may have various implications for the training literature and industry. Commercial training programmes need to demonstrate useful improvement beyond task-specific strategies which are unlikely to benefit the user in their everyday life. Also, confirming that the trained task and outcome measures are structurally different—ideally by demonstrating far-transfer to several different reasoning and intelligence measures—is needed to ensure that transfer effects are not strategy-specific.

Furthermore, older adults may benefit more slowly when attempting to apply a visual strategy—indeed, we found some evidence that implementing the strategy was initially associated with worse performance. While the instructed strategy did appear to somewhat benefit those older adults who were able to apply it (i.e., for maximum, but not average, digit N-back performance), our results did not generalise to the substantial proportion of older adults who chose not to implement (or perhaps were unable to implement) the instructed strategy. Furthermore, older adults spontaneously applied verbal strategies more than did younger adults (with varied success) which suggests differences in spontaneous strategies used by younger and older adults. While our paradigm could not determine if this was driven by preference or ability, it did indicate that perhaps the same training paradigm—or cognitive task, more broadly—is not always measuring the same cognitive capacity in younger and older adults.

The present results highlighted that measures of performance and capacity may largely reflect the extent to which participants apply appropriate strategies, rather than domain-general underlying constructs. Investigating strategies and accounting for individual variability (see Logie, 2018), as well as for systematic, age-related variabilities during real, long-term training, and how specific task strategies may generalise to outcome measures in unintended ways may be essential to resolving discrepancies in the cognitive training literature. On a broader level, the findings are in line with a recently proposed hypothesis, stipulating that the mechanisms underlying WM training are driven by establishment of cognitive routines in the task(s) one has been practicing (which are intertwined with increased strategy use) and that transfer from a trained task (where routine has been established) to an untrained task occurs only if both tasks require the same cognitive routines (Gathercole et al., 2019).

## Supplemental Material

QJE-STD-19-210.R1-Supplementary_Materials – Supplemental material for Strategy mediation in working memory training in younger and older adults Supplemental material, QJE-STD-19-210.R1-Supplementary_Materials for Strategy mediation in working memory training in younger and older adults by Alicia Forsberg, Daniel Fellman, Matti Laine, Wendy Johnson and Robert H Logie in Quarterly Journal of Experimental Psychology

## Notes

1. Power analysis based on the reported effect sizes in Laine et al. (2018): main effect of strategy condition on the trained digit task; $\eta_{\text{p}}^{\text{2}}=.23$ . The weakest significant $\eta_{\text{p}}^{\text{2}}=.15$ in the post-test (untrained letter N-back). To replicate the former (0.95 power) we need 46 younger adults, and the latter (0.80 power) we need 47 younger adults. We recruited 60 participants in each age group to increase power for age comparisons as much as possible within research budget limitations.

2. As only four younger adults reported using rehearsal across the three N-back tasks, we did not include younger adults in these analyses.
