# Supplementary material for: Strategy mediation in working memory training in younger and older adults
Source: Q J Exp Psychol (Hove). 2020 Apr 23;73(8):1206–26. doi: 10.1177/1747021820915107 (PMC7575302; doi:10.1177/1747021820915107)
Supplement: QJE-STD-19-210.R1-Supplementary_Materials – Supplemental material for Strategy mediation in working memory training in younger and older adults [file QJE-STD-19-210.R1-Supplementary_Materials.docx]

**Supplementary Materials for:**

**Strategy Mediation in Working Memory Training in Younger and Older Adults**

Alicia Forsberg, Daniel Fellman, Matti Laine, Wendy Johnson, Robert H. Logie

| Table S1. Participant demographics by age and strategy group (all participants). | | | | | | | |
| --- | --- | --- | --- | --- | --- | --- | --- |
|  | Younger Adults | | | | Older Adults | | |
|  | Control | | Strategy | *p* | Control | Strategy | *p* |
| N | | 29 | 31 |  | 30 | 30 |  |
| Age | | 23.0 (3.96) | 22.0 (2.98) | .29 | 70.3 (5.69) | 68.3 (5.11) | .144 |
| Gender F/M | | 21/8 | 23/8 | 1.0 | 20/10 | 17/13 | .60 |
| Education | | 16.2 (2.81) | 15.7 (2.46) | .55 | 15.5 (3.43) | 16.2 (3.22) | .409 |
| Pre-training N-back composite | | 0.59 (4.94) | -0.23 (5.97) | .577 | 1.04 (4.88) | -1.33 (5.54) | .094 |

*Note.* Values in parentheses are standard deviations. *P*-values were calculated from *t*-tests for continuous variables and χ^2^ test for gender. The N-back composite score represents the summed values of the z-transformations of the average and maximum level accuracy in the adaptive digit N-back task at pretest, and d-prime values and RTs for correct responses in the letter and colour N-back tasks.

Table S2. Mean values (standard deviations) for the pre-post measures per group at pre- and post-test, for younger adults (all participants).

|  | **Control Group (N = 29)** | | | | **Strategy (N = 31)** | | | |
| --- | --- | --- | --- | --- | --- | --- | --- | --- |
|  | **Pre** | **Post** | ***r*** | ***d*** | **Pre** | **Post** | ***r*** | ***d*** |
| **Trained Digit N-back** | | | | | | | | |
| Maximum level | 4.28 (1.71) | 5.52 (2.16) | 0.66 | 0.62 | 3.83 (1.49) | 6.23 (1.98) | 0.47 | 1.35 |
| Average level | 2.72 (0.91) | 3.41 (1.02) | 0.70 | 0.71 | 2.52 (0.94) | 3.84 (1.09) | 0.53 | 1.29 |
| **Untrained N-back Tasks (Task-specific near transfer)** | | | | | | | | |
| Letter 2-back (d-prime) | 2.25 (0.94) | 2.48 (0.96) | 0.71 | 0.24 | 2.12 (1.07) | 2.73 (1.09) | 0.47 | 0.57 |
| Letter 3-back (d-prime) | 1.19 (0.76) | 2.00 (1.15) | 0.55 | 0.80 | 1.09 (1.03) | 2.39 (1.15) | 0.4 | 1.18 |
| Colour 2-back (d-prime) | 2.03 (0.78) | 2.54 (0.93) | 0.39 | 0.59 | 1.92 (1.12) | 2.73 (1.13) | 0.58 | 0.72 |
| Colour 3-back (d-prime) | 0.90 (0.82) | 1.69 (1.22) | 0.52 | 0.74 | 1.02 (0.57) | 2.21 (1.18) | 0.43 | 1.19 |
| Letter 2-back RT (ms) | 803.85 (108.04) | 686.08 (127.44) | 0.51 | -0.99 | 779.64 (141.24) | 647.69 (149.98) | 0.47 | -0.91 |
| Letter 3-back RT (ms) | 802.58 (120.04) | 676.99 (100.28) | 0.36 | -1.13 | 774.63 (213.40) | 633.67 (135.03) | 0.43 | -0.76 |
| Colour 2-back RT (ms) | 811.47 (119.21) | 696.87 (115.34) | 0.26 | -0.98 | 819.43 (122.33) | 662.84 (148.07) | 0.37 | -1.15 |
| Colour 3-back RT (ms) | 857.86 (129.50) | 721.56 (106.42) | 0.24 | -1.15 | 830.50 (227.99) | 674.33 (152.25) | 0.35 | -0.79 |
| **Other Untrained WM Tasks (Task-general near transfer)** | | | | | | | | |
| Selective updating of digits | 32.38 (8.14) | 33.00 (7.08) | 0.78 | 0.08 | 33.42 (9.76) | 35.90 (8.07) | 0.65 | 0.27 |
| Digit span (correct items) | 34.52 (10.00) | 34.10 (8.83) | 0.73 | -0.04 | 33.68 (9.35) | 35.52 (8.97) | 0.38 | 0.20 |
| Digit span (maximum span) | 6.79 (2.06) | 7.28 (1.53) | 0.7 | 0.25 | 7.16 (1.93) | 7.42 (2.05) | 0.24 | 0.13 |
| Running memory | 25.31 (4.49) | 26.28 (5.32) | 0.49 | 0.19 | 23.94 (5.20) | 26.32 (5.93) | 0.57 | 0.43 |
| *Note.* Exclusions to specific analyses apply. | | | | | | | | |

Table S3. Mean values (standard deviations) for the pre-post measures per group at pre- and post-test, for older adults (all participants).

|  | **Control Group (N = 30)** | | | | **Strategy (N = 30 )** | | | | |
| --- | --- | --- | --- | --- | --- | --- | --- | --- | --- |
|  | **Pre** | **Post** | ***r*** | ***d*** | **Pre** | **Post** | ***r*** | ***d*** |  |
| **Trained Digit N-back** | | | | |  |  |  |  |  |
| Maximum level | 3.10 (0.92) | 3.83 (1.29) | 0.51 | 0.64 | 2.77 (0.68) | 3.63 (1.22) | 0.39 | 0.84 |  |
| Average level | 1.94 (0.55) | 2.55 (0.76) | 0.58 | 0.89 | 1.90 (0.41) | 2.40 (0.74) | 0.55 | 0.77 |  |
| **Untrained N-back Tasks (Task-specific near transfer)** | | | | | | | | |  |
| Letter 2-back (d-prime) | 1.85 (0.79) | 2.31 (0.86) | 0.63 | 0.55 | 1.65 (0.89) | 1.98 (0.86) | 0.38 | 0.38 |  |
| Letter 3-back (d-prime) | 0.76 (0.48) | 1.28 (0.88) | 0.45 | 0.68 | 0.70 (0.59) | 1.28 (0.88) | 0.48 | 0.75 |  |
| Colour 2-back (d-prime) | 1.81 (0.75) | 2.09 (0.83) | 0.53 | 0.35 | 1.36 (0.81) | 1.92 (0.96) | 0.43 | 0.62 |  |
| Colour 3-back (d-prime) | 0.77 (0.58) | 0.94 (0.76) | 0.16 | 0.24 | 0.51 (0.50) | 0.86 (0.76) | 0.42 | 0.52 |  |
| Letter 2-back RT (ms) | 1017.30 (165.77) | 869.92 (178.94) | 0.82 | -0.85 | 1000.64 (179.96) | 918.08 (151.49) | 0.51 | -0.49 |  |
| Letter 3-back RT (ms) | 1002.24 (174.61) | 936.59 (167.59) | 0.70 | -0.38 | 996.27 (170.11) | 932.69 (161.45) | 0.67 | -0.38 |  |
| Colour 2-back RT (ms) | 1013.51 (166.92) | 909.24 (160.31) | 0.64 | -0.64 | 1040.48 (161.13) | 948.80 (145.40) | 0.8 | -0. |  |
| Colour 3-back RT (ms) | 1071.56 (160.84) | 959.52 (199.16) | 0.54 | -0.61 | 1023.64 (177.20) | 995.01 (144.42) | 0.45 | -0.18 |  |
| **Other Untrained WM Tasks (Task-general near transfer)** | | | | | | | | |  |
| Selective updating of digits | 24.63 (11.48) | 30.43 (11.33) | 0.75 | 0.51 | 24.50 (13.14) | 26.33 (13.16) | 0.83 | 0.14 |  |
| Digit span (correct items) | 33.23 (8.24) | 34.37 (7.91) | 0.64 | 0.14 | 32.17 (8.73) | 33.63 (8.19) | 0.63 | 0.17 |  |
| Digit span (maximum span) | 6.93 (1.36) | 7.23 (1.36) | 0.18 | 0.22 | 6.67 (2.04) | 6.83 (1.72) | 0.54 | 0.09 |  |
| Running memory | 24.33 (4.33) | 23.80 (5.29) | 0.51 | -0.11 | 22.67 (5.14) | 23.83 (4.61) | 0.59 | 0.24 |  |
| *Note.* Exclusions to specific analyses apply. | | | | | | | | |  |

Table S4. ANCOVA results for the trained task and for the transfer measures (all participants).

|  |  | ***F*** | ***p*** | ***d* / *ηp^2^*** |
| --- | --- | --- | --- | --- |
| **Trained Digit N-back** | | | | |
| Maximum level | Strategy | 4.17 | .065 | 0.37 |
|  | **Age** | **20.67** | **<.001** | **0.87** |
|  | Interaction | 3.58 | .073 | 0.03 |
| Average level | Strategy | 2.85 | .094 | 0.30 |
|  | **Age** | **17.86** | **<.001** | **0.81** |
|  | **Interaction** | **6.74** | **.021** | **0.06** |
| **Untrained N-back Tasks (Task-specific near transfer)** | | | | |
| Letter 2-back (d-prime) | Strategy | 0.13 | .785 | 0.07 |
|  | Age | 2.23 | .268 | 0.28 |
|  | Interaction | 3.4 | .194 | 0.03 |
| Letter 3-back (d-prime) | Strategy | 2.15 | .268 | 0.27 |
|  | **Age** | **14.38** | **.001** | **0.71** |
|  | Interaction | 1.47 | .365 | 0.01 |
| Colour 2-back (d-prime) | Strategy | 1 | .466 | 0.19 |
|  | **Age** | **6.54** | **.041** | **0.49** |
|  | Interaction | 0.29 | .679 | <.001 |
| Colour 3-back (d-prime) | Strategy | 2.27 | .268 | 0.29 |
|  | **Age** | **22.42** | **<.001** | **0.90** |
|  | Interaction | 0.96 | .466 | 0.01 |
| Letter 2-back (RT in ms) | Strategy | 0.58 | .597 | 0.14 |
|  | **Age** | **11.61** | **.004** | **-0.68** |
|  | Interaction | 3.28 | .194 | 0.03 |
| Letter 3-back (RT in ms) | Strategy | 0.52 | .598 | -0.13 |
|  | **Age** | **49.98** | **<.001** | **-1.39** |
|  | Interaction | 0.45 | .607 | <.001 |
| Colour 2-back (RT in ms) | Strategy | 0.09 | .785 | -0.06 |
|  | **Age** | **21.83** | **<.001** | **-0.96** |
|  | Interaction | 1.94 | .285 | 0.02 |
| Colour 3-back (RT in ms) | Strategy | 0.07 | .785 | 0.05 |
|  | **Age** | **45.07** | **<.001** | **-1.34** |
|  | Interaction | 2.82 | .23 | 0.03 |
| **Other Untrained WM Tasks (Task-general near transfer)** | | | |  |
| Selective updating of digits | Strategy | 0.59 | .799 | -0.14 |
|  | Age | <.001 | .976 | 0.01 |
|  | Interaction | 6.51 | .145 | 0.05 |
| Digit span (correct items) | Strategy | 0.47 | .799 | 0.13 |
|  | Age | <.001 | .976 | 0.01 |
|  | Interaction | 0.64 | .799 | 0.01 |
| Digit span (maximum span) | Strategy | 0.28 | .799 | -0.10 |
|  | Age | 0.77 | .799 | 0.16 |
|  | Interaction | 0.28 | .799 | <.001 |
| Running memory | Strategy | 1.29 | .799 | 0.21 |
|  | **Age** | 4.82 | .181 | 0.40 |
|  | Interaction | 0.01 | .976 | <.001 |
|  |  |  |  |  |

*Note.* To correct for multiple comparisons, Benjamini-Hochberg adjusted p-values were applied for group comparisons on each pre-post outcome measure.

| Table S5. Classification scheme for strategy types based on participants’ self-reports at post-test, used by independent raters.  **CODING CRITERIA: STRATEGY CLASSIFICATION** | |
| --- | --- |
| **Scoring** | **Example** |
| **Rehearsal (1)** | - “I repeated the digits silently in my mind” - “I repeated a list of letters in my mind” - “Repeating out loud the letters” |
| **Grouping (2)** | - “I created groups of 3 digits” - “I grouped the letters in pairs” - “When the sequence was long enough I remembered the items in groups of 4” - “I remembered the digits in groups” |
| **Updating (3)** | - “I created a group of digits in my mind and dropped the last digit when a new digit appeared” - “Replaced the first letter with the latest letter that appeared on the screen.” - “I tried to replace each color one at a time as a new color came up” |
| **Grouping and comparison (4)** | - “I split the digits into different series, and compared those to each other” - “Held each sequence of digits in mind, removed the first and added a new digit to the end. After that, I checked if it was the same as the one just dropped.” |
| **Semantics (5)** | - “I created words from the letters (e.g., C-R-S = Corn – Rose – Sand)” - “I converted the digits to melodies, e.g., 1356 = DO-MI-SO-LA” |
| **Phonology (6)** | - “I made up lists based on initial parts of the digit names such as se-fi-ni (7-5-9)” - “I tried to make syllables out of the letters” |
| **Imagery (7)** | - “I tried to associate each digit with some image in my mind” - “I tried to visualize the letters as snakes” |
| **Visualization (8)** | - “I visualized the numbers” - “I tried to visualize the letter sequence in my mind” |
| **Familiarity (9)** | - “I chose the letters that felt most familiar” - “I recalled the digits that were familiar” |
| **Guessing (10)** | - “I just used intuition” - “I started somewhere in the middle of the sequence, and did not memorize the first digits in the sequence at all” |
| **Other strategies (11)** | - “I made up a song based on the letters” - “Yes” - “I tried to keep all the digits in my mind” - “This task was difficult” |
| **No strategy use (12)** | - “I pressed the N-key if the current white box was the same as the white box presented before it, or the M-key if it was not the same” |

| Table S6. The frequency of occurrence (in percent) of reported strategy types by the control participants. | | | | | | | |
| --- | --- | --- | --- | --- | --- | --- | --- |
|  | *N-back Digit* | | | *N-back Letter* | | *N-Back Colour* | |
|  | Younger | | Older | Younger | Older | Younger | Older |
| Rehearsal | | 3.45 | 31.03 | 6.90 | 27.59 | 3.45 | 24.14 |
| Grouping | | 13.79 | 10.34 | 10.34 | 13.79 | 10.34 | 3.45 |
| Updating | | 0.00 | 0.00 | 0.00 | 0.00 | 3.45 | 0.00 |
| Grouping and comparison | | 10.34 | 0.00 | 13.79 | 0.00 | 3.45 | 0.00 |
| Semantics | | 0.00 | 0.00 | 3.45 | 0.00 | 0.00 | 0.00 |
| Phonology | | 0.00 | 0.00 | 0.00 | 0.00 | 0.00 | 0.00 |
| Imagery | | 0.00 | 0.00 | 0.00 | 0.00 | 0.00 | 0.00 |
| Visualization | | 3.45 | 3.45 | 0.00 | 0.00 | 0.00 | 3.45 |
| Familiarity | | 0.00 | 0.00 | 0.00 | 0.00 | 0.00 | 0.00 |
| Guessing | | 0.00 | 0.00 | 0.00 | 0.00 | 0.00 | 0.00 |
| Other strategies | | 27.59 | 17.24 | 13.79 | 6.90 | 17.24 | 13.79 |
| No strategy use | | 41.38 | 37.93 | 51.72 | 51.72 | 62.07 | 55.17 |

| Table S7. Level of Detail rating instructions for strategies. The examples were created as an aid for the independent raters.  **CODING CRITERIA: LEVEL OF DETAIL** | | |
| --- | --- | --- |
| **Scoring** | **Description** | **Example responses** |
| **No strategy use (0)** | *Ticks the “No” –alternative and has no comments in the text box.*  *OBS! If the participant has commented anything on his/her strategy use of in the comment box, yet ticked "No", make your evaluation based on the comment. In other words, if the comment shows that the participant has used some kind of strategy, he/she can get points (depending on the content of the comment).* |  |
| **Yes-alternative + non-specific description of strategy (1 point)** | *There is not a clear strategy in the comment. The comment may be short or long, but the description of the strategy is non-specific. The participant may also repeat the task instructions provided at the beginning of each pre- and posttest task (i.e., there is nothing new in the comment except for the instructions that already has been provided).*   - **Examples:** - Only writes that he/she tried to remember the stimuli in his/her head/mind/read aloud but does not describe how. - Writes that he/she has acted on intuition - Reports that he/she did not use any consistent strategy - Reports that he/she used different strategies depending on the situation. | - “I memorized the digits in my mind” - “I used intuition” - “I did not use any strategy consistently” - “I used different strategies depending on the situation” - “I tried to keep the items in my head” - “I tried to remember the digits in my head” - “I read the colors aloud” - ”I said the numbers aloud” |
| **Yes-alternative + general strategy description (2 points)** | *The comment clearly indicates that the participant has used a strategy (i.e., he/she writes the way in which the strategy was used, not only that "I tried to remember / keep the items in my head / read aloud") but the description of the strategy remains at a* *general level.*   - **Examples** - Writes that he/she repeated the stimuli series in his/her mind. - Writes that he/she read the items in series in his/her mind. - Writes that he/she used e.g., rhythm, grouping, fingers to memorize the items. | - “I repeated the letters in my mind in two or three groups” - “I memorized the digits in pairs, such as 52-48” - “Grouping the digits in separate series” |
| **Yes-alternative + two or more details of the used strategy in addition to the general strategy description (3 points)** | *The comment clearly indicates that the participant have used a strategy, and describes related details of the strategy.*   - **Example:** - Writes that he/she split the stimuli series into separate chunks and compared them to each other. | - ”I tried to keep 2 numbers back in my mind while saying 1 number back out loud to try to keep in my mind the 2 differing numbers.”, - “Memorized the four letters as one sequence and replaced each letter as a new one came up”   “I split the digits into different series, and compared those to each other” |

### Motivation and Expectations (also including non-compliant participants)

### Below, we report analyses conducted including all participants (i.e., also including those strategy-group participants who reported not complying with the strategy instruction).

### Alertness, motivation, and expectations. Participants’ expected improvement during training was probed right before they started the training, to assess if receiving a strategy would make a difference. There was no difference in expectation between strategy and control groups in younger (*t*(55.09) =  0.08, *p*  = .94) or older adults *t*(56.38) =  0.56, *p* = .58). We also compared expected improvement on each task between pre-test and post-test, after the strategy group had received the strategy instruction. There were no significant differences on any of the tasks, in either age group (all p-values ≥ .13). Similarly, there we no differences in self-reported alertness or motivation in either age group just after they completed the training session (all p-values ≥ .20).

### Training Improvement. Two independent samples t-tests found no overall effect of Strategy Instruction in the younger (*t*(58) =  0.88, *p*  = .38, *d* = 0.23; strategy group *M* = 4.29digits, control group *M* = 3.93 digits), or in the older adults (*t*(58) =  -0.39, *p*  = .70, *d* = 0.10, strategy group *M* = 2.48 digits, control group: *M* = 2.58 digits) on average performance across all 20 trials.

### Pre-test Composite Scores: Compliant vs Non-Compliant Strategy group participants

### Two exploratory independent samples t-tests found no differences in pre-test composite score between compliant and non-compliant participants in the younger (*t*(26) =  -0.002, *p*  = .999, *d* = -0.001), or in the older adults (*t*(25) =  -0.161, *p*  = .873, *d* = -0.064). The pretest composite score comprised of the summed values of the z-transformations of the average and maximum level accuracy in the adaptive digit N-back task, and d-prime values and RTs for correct responses in the letter and colour N-back tasks.
